# Impact of Rural Sanitary Toilet Interventions on pesticide reduction: health literacy as a mediating effect

**DOI:** 10.3389/fpubh.2026.1793384

**Published:** 2026-03-19

**Authors:** Nian Zhang, Xiangxiong Xiao, Dehua Li

**Affiliations:** School of Public Administration, Xiangtan University, Xiangtan, Hunan, China

**Keywords:** health literacy, pesticide reduction, Rural Development, Sanitary toilets, sustainable agriculture

## Abstract

**Introduction:**

China's Rural Sanitary Toilet Interventions (2015-present) is a government-led initiative aimed at replacing unhygienic rural toilets with sanitary facilities. This study explores the impact of this policy on pesticide reduction and analyzes the mediating role of health literacy.

**Methods:**

Based on survey data from 4,277 rural households in 10 provinces, this study employed a three-step method and bootstrap resampling for mediation analysis, used alternative models for robustness checks, and conducted heterogeneity analysis.

**Results:**

The significant reduction in pesticide use due to the Rural Sanitary Toilet Interventions project remained robust across different model specifications. Heterogeneity analysis revealed that the policy had a particularly pronounced effect on reducing pesticide use in the lower-middle-income group, while no significant differences were observed in other income groups. The effect was significant in non-plain areas but not statistically significant in plain areas. Mediation analysis confirmed that health literacy played a crucial mediating role in the relationship between policy implementation and the reduction in pesticide use.

**Discussion:**

These findings emphasize the importance of tailoring policies to different income levels and geographical conditions for targeted interventions, as well as the necessity of integrating health education with agricultural practices to promote sustainable agricultural behaviors. This study contributes to the literature by bridging the gap between public health and agricultural sustainability, offering valuable insights for policymakers seeking to promote eco-friendly agricultural transitions.

## 1 Introduction

Pesticide reduction is a crucial component of the United Nations Sustainable Development Goals (SDGs) (1), particularly Target 2 (Zero Hunger) and Target 15 (Life on Land), which emphasize sustainable agricultural practices and biodiversity conservation. The 34th session of the United Nations Human Rights Council noted with concern that approximately 200,000 people die from pesticide poisoning annually, with a staggering 99% of these deaths occurring in developing countries. As a major agricultural country and a developing nation, China accounts for 10.27% of global pesticide usage. However, its pesticide utilization efficiency is only 40.6%, leading to contamination of 10 million hectares of farmland and an annual grain production loss exceeding 10 million tons due to soil pollution (2). Pesticides not only cause severe damage to soil and ecosystems but also pose serious health risks, including cancer, neurological disorders, and infertility (3–5). Vulnerable groups, especially women and children, are disproportionately affected, undermining their health and wellbeing (6). Therefore, promoting pesticide reduction has become an urgent imperative for achieving agricultural sustainability and rural ecological objectives.

China's Rural Sanitary Toilet Interventions (hereafter abbreviated as RSTI) is a government-led initiative launched in 2015, aimed at replacing unsanitary rural toilets with hygienic facilities. In November 2017, the Chinese government further prioritized this “RSTI” as a key measure to improve rural living environments in the coming years. This policy is of significant importance for enhancing the quality of life, safeguarding fundamental human rights (7), protecting the ecological environment (8), and advancing social civilization (9). The use of sanitary toilets yields substantial health benefits in terms of pollution control and rural environmental improvement (10). It not only improves residents' health standards and reduces the disease burden, but also increases school enrollment rates, contributing to holistic human capital development (11–15). However, the impact of sanitary toilets on the health of rural residents exhibits significant regional disparities (16). Individual orthodox beliefs regarding cleanliness and pollution also act as barriers (17). As primary victims of inadequate sanitation facilities, women are particularly sensitive to their safety (18), and the effects of sanitary toilets usage are more pronounced among women, middle-aged individuals, and low-income groups (19). Existing research notes the policy's “spillover effect,” which enhances community members' environmental awareness and generates unintended positive impacts in other areas (20). Against this backdrop, this study aims to explore, at the micro level, the impact mechanisms and transmission pathways through which RSTI influences pesticide reduction.

Health literacy serves as a crucial link between health-related cognitive understanding and actual practices, playing a pivotal role in promoting individual health behaviors. The Health Belief Model (HBM) and the Theory of Planned Behavior (TPB) provide complementary frameworks for explaining this mechanism. Within the HBM framework, the health education accompanying RSTI acts as a powerful cue to action. This enhances farmers' health literacy, enabling them to better perceive the health risks of pesticide overuse and the benefits of reduction. Consequently, they are more likely to adopt environmentally friendly practices after weighing perceived barriers such as yield concerns, technical challenges, and transition costs (21–24). The TPB further elucidates how this translated cognition drives behavior. Improved health literacy reshapes farmers' attitudes by highlighting the trade-off between short-term yields and long-term health/ecological costs. Simultaneously, as a community-wide intervention, RSTI strengthens subjective norms by fostering pro-environmental consensus within villages. It may also enhance perceived behavioral control through organizational support (e.g., training, subsidies), thereby strengthening farmers' intention and ability to reduce pesticide use (25). Therefore, health literacy constitutes a critical mediating pathway through which RSTI influences pesticide-reduction behaviors.

Existing research on the health-environment spillover effect has established a relatively mature analytical framework, primarily focusing on verifying the association between “abstract health interventions” and “environmental behaviors,” with most studies confirming that increased health awareness has a positive spillover effect on environmental behaviors (26–28). Compared to previous research, this study makes marginal contributions in three aspects: first, this study is the first to interdisciplinary link “RSTI” in public health with “pesticide reduction” in agricultural environmental policies, offering a novel perspective for understanding how health-related policies influence agricultural green transformation. Second, it reveals the internal mechanism through which RSTI indirectly promotes pesticide reduction by enhancing farmers' health literacy, clarifying the complex interaction between human behavior and agricultural production in the context of sustainable agricultural transformation. Third, it finds that the policy effect is more significant in non-plain areas and among lower-middle-income groups, challenging the traditional linear assumption that “economic level determines environmental behavior” and resonating with the grassroots empowerment perspective of Environmental Justice Theory.

The remainder of the paper is structured as follows: section two presents theoretical analysis and data samples; section three reports the empirical results; section four discusses these findings; and section five presents the conclusions and policy implications.

## 2 Methodology

### 2.1 Direct Effect of RSTI on pesticide reduction

Firstly, at the level of direct environmental effects, the RSTI has improved rural toilet environments, promoting the harmless treatment and resource utilization of feces. Traditional unhygienic toilets are prone to becoming breeding grounds for pathogens and pests, which may migrate to farmland, leading to an increase in crop pests and diseases, thereby prompting farmers to increase pesticide usage. However, sanitary toilets can block this transmission route, reducing the probability of pests and diseases occurring. The production of harmless organic fertilizer through feces fermentation mitigates the direct pollution of feces as a contaminant to soil and water bodies, which is conducive to maintaining the balance of agricultural ecology, promoting healthy crop growth, and reducing dependence on pesticides.

Furthermore, at the level of behavioral spillover effects, RSTI is a nationally led public health initiative with clearly defined norms. Its implementation not only transforms the physical environment but also fosters a new community norm centered on hygiene and health awareness. The strong subjective norms driven by policy implementation and community participation, together with the positive experiences brought about by the improved environment, collectively trigger broad adjustments in individual behavior. According to behavioral spillover theory, pro-environmental and health-oriented behavioral tendencies developed in one domain can naturally extend to other related domains (29, 30). Thus, after complying with new hygiene regulations and experiencing their health benefits, farmers are more likely to extend their pursuit of “cleanliness” and “safety” to field management, actively adopting more ecologically beneficial production practices, such as reducing chemical pesticide use and implementing green pest control technologies.

In summary, RSTI not only creates objective conditions for pesticide reduction through direct ecological and environmental improvements but also subjectively motivates farmers to reduce pesticide use through the behavioral norms and psychological spillover effects generated by policy intervention. On this basis, this paper proposes Hypothesis 1:

**Hypothesis 1:** RSTI has a significant positive impact on pesticide reduction.

### 2.2 Mediating the role of health literacy

Health literacy refers to the personal knowledge and abilities accumulated through daily activities, social interactions, and intergenerational transmission, which has been recognized as a key factor influencing health outcomes and behavioral choices (31). As a critical psychological and cognitive link connecting sanitary environment interventions and agricultural production behaviors, the mediating role of health literacy can be explained through the intersecting perspectives of environmental behavior theory, environmental justice theory, the Health Belief Model, and the Theory of Planned Behavior.

From the perspective of the Health Belief Model and its connection to health literacy, RSTI is not merely infrastructural upgrades but is accompanied by mechanisms to enhance health literacy, such as health education training, social interaction-based dissemination, and media campaigns—which help farmers accumulate knowledge and skills related to environmental health and health risks, thereby gradually enhancing their health literacy. Enhanced health literacy, in turn, strengthens farmers' perception of the environmental damage and health hazards caused by pesticide use, enabling them to more clearly recognize the negative consequences of pesticide overuse. Integrated with the Theory of Planned Behavior, farmers with higher health literacy are better able to accurately discern the subjective norms and perceived behavioral control conditions associated with “reducing pesticide use,” thereby strengthening their willingness and capacity to adopt pesticide reduction behaviors.

From the standpoint of environmental behavior theory, improved health literacy encourages farmers to extend the “health-environment” linkage established through RSTI to the agricultural production domain. The higher the level of health literacy, the more inclined farmers are to integrate personal health behaviors with public environmental benefits, leading them to choose greener and more environmentally friendly agricultural practices, such as reducing chemical pesticide use. From an environmental justice perspective, RSTI is itself a measure to improve the equity of environmental resources in rural areas, providing farmers with safer sanitary conditions. Enhanced health literacy makes farmers more aware of their own and their community's right to equitable access to ecological resources, motivating them to actively engage in pesticide reduction to maintain the sustainability of agricultural ecosystems, thereby achieving dual benefits for health and ecological integrity.

In summary, RSTI enhances farmers' health literacy through supporting mechanisms, and this improved health literacy, in turn, promotes pesticide reduction behaviors through cognitive, motivational, and behavioral pathways. On this basis, this paper proposes Hypothesis 2:

**Hypothesis 2:** RSTI increases health literacy, thereby enhancing pesticide reduction.

**Figure 1:**
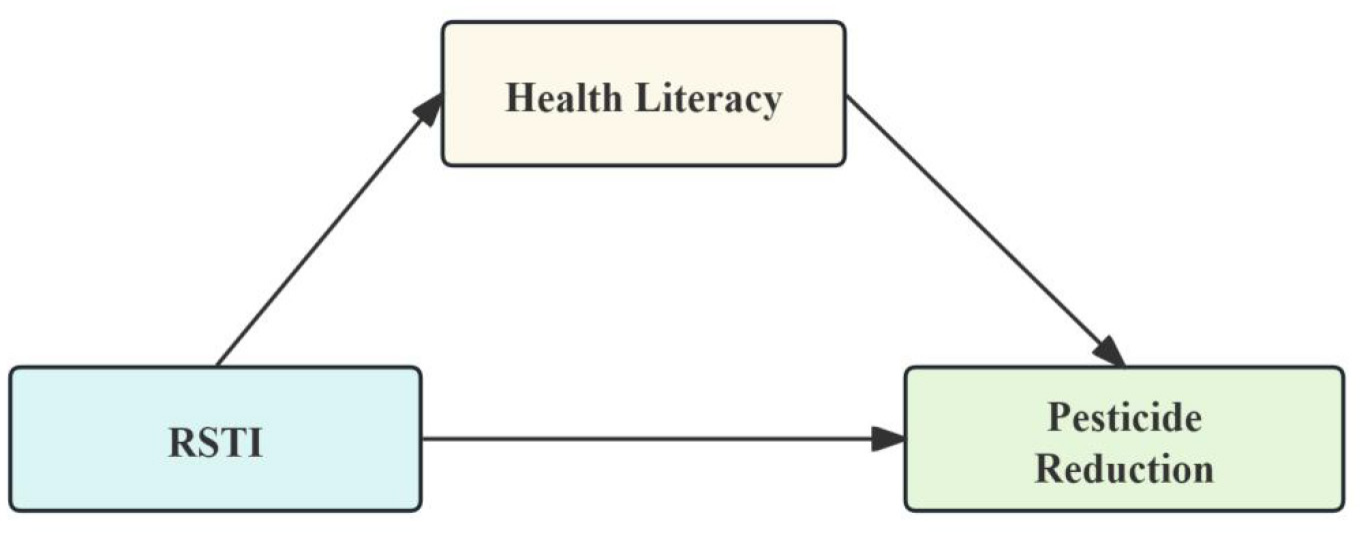
Proposed conceptual model for this study.

Based on the above theoretical analysis, a theoretical model illustrating the relationship between RSTI, health literacy, and pesticide reduction was developed (Figure 1).

### 2.3 Data source and samples

The data used in this study were obtained from the 2020 CRRS, which was conducted by Rural Development Institute, Chinese Academy of Social Sciences in 2020. To ensure a representative sample, the sampling process was designed in multiple stages, taking into account regional diversity, economic development levels, and geographic distribution. First, 10 provinces were purposively selected from the eastern, central, and western regions of the country based on their economic development status, geographic location, and agricultural production characteristics. This ensured a balanced representation of China's diverse socioeconomic and geographic contexts. Second, within each selected province, all counties (including cities and districts) were stratified into five groups according to per capita GDP. To ensure geographic representativeness, one county was randomly selected from each group, resulting in five counties per province. This stratified random sampling approach aimed to capture variations in economic development across different regions. Third, following a similar stratification principle, three townships were randomly selected from each county to represent high, medium, and low levels of economic development. Within each township, two administrative villages were further selected to reflect high and low economic development levels, respectively. This multi-stage sampling strategy ensured that both inter-regional and intra-regional heterogeneity were adequately captured. Finally, within each administrative village, 12 to 14 households were selected using systematic sampling based on rosters provided by local village committees. These households were surveyed using a structured questionnaire. Through this rigorous sampling methodology, the project team covered a total of 10 provinces, 50 counties, 156 townships, and 308 administrative villages. More than 15,000 household members were surveyed, providing a robust dataset for analysis. After excluding cases with missing data for relevant variables, the final dataset comprised 4,277 valid cases, which were used for analysis.

### 2.4 Variables and measurement

#### 2.4.1 Pesticide reduction

The average pesticide usage cost per unit planting area of farmland households is used as an indicator for representation. The questionnaire inquired about the expenses incurred in purchasing insecticides, herbicides, and disease-control medications. To mitigate the impact of heteroscedasticity, these expense data were log-transformed. Pesticide Reduction is measured by the average pesticide expenditure per unit planting area, reflecting changes in farmers' economic decisions regarding pesticide inputs. Farmers' record-keeping typically centers on “expenditure amount” and “planting area,” making this metric a direct reflection of the core dimension of farmers' input decisions. From the perspective of pesticide reduction policy practice, “changes in input cost” also carry practical significance, as policymakers are often more concerned with whether reduction measures translate into perceptible cost-benefit changes for farmers. Therefore, in the absence of systematic data on actual pesticide usage or toxicity levels, pesticide expenditure remains a widely used and valid indicator for assessing changes in farmers' pesticide input behavior. It should be noted that changes in pesticide expenditure may be influenced by non-quantity factors (e.g., price fluctuations, substitution among pesticide types), which we further discuss in the limitation section.

#### 2.4.2 RSTI

Whether a household has a sanitary toilet with harmless waste treatment is used as a proxy variable for RSTI. The response options are “Yes” and “No”, which are coded as 1 and 0, respectively.

This measurement is scientifically justified and policy-relevant, especially supported by the strict definition and standardized annotation in the CRRS questionnaire: the survey explicitly defines a “sanitary toilet with harmless waste treatment” as “a facility with walls, a roof, a door, a standard toilet bowl inside, clean without maggots and flies, basically odorless, and equipped with a septic tank that achieves harmless treatment of feces—without leakage, seepage, and with a sealed cover”. During the survey, this annotation was fully communicated to respondents, ensuring that all participants clearly understood the quality and functional standards of a qualified “sanitary toilet” before answering. Thus, the binary variable of “having or not having such a toilet” is not a simplistic distinction of “presence or absence”, but rather a quality-screened indicator that implicitly includes unified standards for infrastructure completeness, hygiene conditions, and environmental safety.

Furthermore, this measurement aligns with the core goal of the RSTI policy—promoting the popularization of “hygienic and harmless” toilets rather than merely increasing the quantity of toilets. Using this indicator directly reflects whether a household has effectively participated in the policy intervention, and its objectivity (avoiding subjective judgment of toilet quality by researchers) and consistency (uniform standards across all samples) make it a suitable proxy for examining the spillover effect from sanitation improvement to agricultural practices.

#### 2.4.3 Health literacy

The 2020 CRRS inquiries from respondents, “Do you consciously seek knowledge about health or wellness?” Responses were coded such that “Yes” was assigned a value of 1, while “No” was assigned a value of 0.

This study focuses on the “cognitive-behavioral link” between public health interventions (Rural Sanitary Toilet Interventions, RSTI) and agricultural production behaviors (pesticide reduction). The question “Do you consciously seek health-related knowledge?” directly captures farmers' proactive attention to health issues—a foundational and core behavioral manifestation of health literacy. This indicator aligns perfectly with the proposed transmission pathway of “RSTI → enhanced health awareness → pesticide reduction,” as it targets the policy-driven critical cognitive shift: from passive acceptance of health information to active pursuit of it.

From the perspective of research objectives, this study aims to examine whether health literacy plays a mediating role between RSTI and pesticide reduction, rather than conducting a comprehensive, multidimensional measurement of farmers' health literacy. Selecting the dimension most relevant to the core transmission mechanism—active health information seeking—can most directly capture the cognitive changes induced by the policy intervention, avoiding measurement noise from dimensions unrelated to this pathway. From the perspective of data realities, the 2020 CRRS is a comprehensive panoramic survey on rural revitalization rather than a specialized investigation of health literacy, and thus did not incorporate a standardized multidimensional health literacy scale. Under this constraint, the single indicator employed represents the measurement tool closest to the theoretical construct and most operable within the existing dataset. Regarding indicator validity, the behavior of actively seeking health knowledge is precisely one of the core outcomes that accompanying policy initiatives—such as health education campaigns and community mobilization—aim to achieve. This indicator directly reflects the operationalized outcome of the policy intervention at the cognitive level, specifically whether the policy has genuinely motivated farmers to engage with health-related information.

It must be acknowledged that employing a single binary indicator cannot comprehensively capture the multidimensional nature of health literacy, including its functional, interactive, and critical dimensions. In the conclusion section, we discuss this limitation in detail and propose that future research should incorporate standardized multidimensional health literacy scales (such as the short version of the HLS-EU-Q) to measure this construct more precisely and comprehensively, thereby further validating and deepening the findings of this study.

#### 2.4.4 Control variables

To account for potential confounders, the model includes key individual and land management characteristics that may influence pesticide use decisions, making it necessary to include them as control variables in the analysis. At the individual farmer level, age and gender may influence knowledge and adoption of modern agricultural practices. Education level can affect farmers' understanding of pesticide use and environmental concerns. Political affiliation, marital status, and health status can also influence farmers' access to information, resources, and support networks, which in turn may affect their pesticide use. At the land management characteristics, the proportion of grain used for self-consumption may indicate farmers' reliance on their own produce and thus their motivation to ensure high quality and safety. Net income from farming reflects the economic incentives and constraints faced by farmers in adopting sustainable practices. It should be noted that in Table 1, LnIPA represents the logarithm of the average net income from crop farming per unit planting area. LnECA represents the logarithm of the average ecological compensation fee received per unit planting area. Farmers who receive ecological compensation may have a stronger incentive to reduce pesticide use in order to comply with the requirements of the compensation policy. In summary, to isolate the effects of the primary variables of interest while accounting for potential confounding factors, this study controls for key individual and land management characteristics. This approach enhances the robustness of the findings and yields meaningful insights into the determinants of pesticide application practices among farmers.

**Table 1:** Description and descriptive statistics of research variables.

**Variable category**	**Variable name**	**Variable coding**	**Mean**	**Standard error**
Explained variable	LnPR	Logarithm of average pesticide usage cost per unit planting area (¥·hm^2^)	3.27172	1.98860
Explanatory variable	RSTI	Does your household have a sanitary toilet with harmless waste treatment? (1 = Yes,0 = No)	0.72364	0.44725
Individual characteristics	Gender	Respondent's gender (1 = Male, 0 = Female)	0.52560	0.49940
	Age	Measured in years	41.09843	21.13770
	Education level	Variable assignment (0 = Illiterate, 1 = Primary school, 2 = Middle school, 3 = High school, 4 = College, 5 = Bachelor's degree or above)	1.70143	1.13038
	Marital status	Marital status (1 = Married, 0 = Unmarried)	0.66729	0.47124
	Political affiliation	Respondent's political affiliation (1 = Communist party member, 0 = Non-member)	0.08721	0.28218
	Health status	Health status compared to peers (1 = Very poor, 2 = Poor, 3 = Fair, 4 = Good, 5 = Very good)	3.53262	1.04692
Land management characteristics	Self-consumption ratio	The proportion of grain crops produced for household consumption	24.85172	38.44121
	LnIPA	Logarithm of average net income from crop farming per unit planting area (¥·hm^2^)	5.41146	3.08925
	LnECA	Logarithm of average ecological compensation fee per unit planting area (¥·hm^2^)	1.09264	2.25403

¥**·**hm^2^ denotes Chinese Yuan per hectare.

### 2.5 Model specifications

To examine the direct effect of RSTI on pesticide reduction, we estimate the following multiple regression model corresponding to Hypothesis 1:


$$\begin{array}{c} LnPR_{i}=\beta_{0}+\beta_{1}RSTI_{i}+\beta^{T}X_{i}+\varepsilon_{i} \end{array}$$ (1)

Where:

LnPR_*i*_ denotes the logarithm of pesticide expenditure per unit planting area for household *i*, serving as the proxy for pesticide reduction.

RSTI_*i*_ is a binary indicator for Rural Sanitary Toilet Interventions (1 = has sanitary toilet; 0 = otherwise).

β_1_ is the coefficient of the core explanatory variable RSTI.

X_*i*_ is a vector of control variables, including household head characteristics (age, education level, marital status, political affiliation, health status) and household/farm management characteristics (self-consumption ratio, net farm income per unit area, ecological compensation, etc.).

ε_*i*_ is the error term.

To explore the role of health literacy in the influence that the RSTI has on pesticide reduction, Hypothesis 2 was tested and the following model was constructed:


$$\begin{array}{c} HealthLit_{i}=a_{0}+a_{1}RSTI_{i}+a^{T}X_{i}+u_{i} \end{array}$$ (2)

$$\begin{array}{c} LnPR_{i}=\delta_{0}+\beta_{1}RSTI_{i}+\theta_{1}HealthLit_{i}+\delta^{T}X_{i}+v_{i} \end{array}$$ (3)

Where:

HealthLit_*i*_ is the mediator, a binary measure of health literacy (1 = actively seeks health knowledge; 0 = otherwise).

In Equation (2), coefficient α_1_ estimates the effect of RSTI on health literacy.

In Equation (3), coefficient θ_1_ estimates the effect of health literacy on pesticide expenditure, and coefficient β_1_ estimates the direct effect of RSTI after controlling for the mediator.

*u*_*i*_ and *v*_*i*_ are error terms.

## 3 Results

### 3.1 Multicollinearity test

To test the problem of multicollinearity among the variables and to ensure the scientific rigor of the results of the next step of the analysis, the Variance Inflation Factor (VIF) test was carried out. As shown in Table 2, the VIF of each variable is less than 2, and the mean value of VIF is 1.2, which indicates that there is no serious multicollinearity problem among the variables, and the next step of analysis can be carried out.

**Table 2:** Multicollinearity test.

**Variable**	**VIF**	**1/VIF**
Age	1.77	0.56613
Marital status	1.71	0.58569
LnIPA	1.12	0.89541
Political affiliation	1.10	0.90828
Education level	1.10	0.91074
Self-consumption ratio	1.09	0.92041
LnECA	1.06	0.94521
RSTI	1.04	0.95733
Gender	1.04	0.96027
Health status	1.02	0.98088
Mean VIF	1.2	

### 3.2 Regression analysis of RSTI and pesticide reduction

Table 3 summarizes the results of the multiple linear regression analysis. Model 1 includes only the RSTI, while Model 2 incorporates all control variables. The results show that, after controlling for relevant variables, the intervention has a significant negative effect on the logarithm of average pesticide usage cost per unit planting area (β = −0.237, *p* < 0.01). This coefficient indicates that RSTI is associated with an average reduction of approximately 21% in pesticide expenditure per unit planting area. These findings suggest that RSTI play a significant role in promoting pesticide reduction, thereby confirming Hypothesis 1.

**Table 3:** Regression results: impact of RSTI on pesticide reduction.

**Variable**	**Model 1**	**Model 2**
	**Pesticide reduction**	**Pesticide reduction**
Constant	3.481^***^	3.000^***^
	(0.058)	(0.160)
RSTI	−0.289^***^	−0.237^***^
	(0.068)	(0.068)
Gender		−0.029
		(0.061)
Age		−0.001
		(0.002)
Education level		−0.001
		(0.028)
Marital status		0.004
		(0.083)
Political affiliation		−0.039
		(0.111)
Health status		0.042
		(0.029)
Self-consumption ratio		−0.005^***^
		(0.001)
LnIPA		0.066^***^
		(0.009)
LnECA		0.001
		(0.011)
Sample size	4,277	4,277
Adjusted R^2^	0.004	0.029
F-value	18.077^***^	13.629^***^

Values in parentheses represent standard errors. ^***^p < 0.01.

### 3.3 Robustness tests

#### 3.3.1 Excluding autonomous regions and economically developed provinces

Ningxia Hui Autonomous Region is the largest Hui-populated area in China, where the Hui people adhere to Islam. Due to the religious and cultural emphasis on “cleanliness”, they may voluntarily promote the reduction of pesticides to avoid the use of “unclean” chemicals. This provides an alternative explanation for the health literacy mechanism underlying the RSTI. In contrast, Guangdong and Zhejiang, characterized by high levels of economic development and strong financial capacity, often bundle the RSTI with pesticide reduction policies, creating a policy synergy that is difficult to disentangle. Therefore, this paper employs samples excluding these three provinces and autonomous regions to re-estimate the impact of the RSTI on pesticide reduction. The results indicate that the RSTI is still associated with a significant reduction in pesticide use cost (Model 3).

#### 3.3.2 Robustness check based on PSM method

To address potential sample self-selection, the baseline model was re-estimated using propensity score matching (PSM) with three alternative algorithms: one-to-one nearest neighbor matching, kernel matching, and radius matching. The results from one-to-one nearest neighbor matching (Model 4) indicate that the coefficient for the RSTI is −0.506, which is significant at the 1% level (^*^p < 0.01). The balance test reveals that the maximum standardized bias among all covariates after matching is 6.6%, suggesting good matching quality. Similarly, the kernel matching (Model 5) results show a coefficient of −0.236 for the intervention, also significant at the 1% level. The maximum standardized bias post-matching is 5.3%. The radius matching (Model 6) yields a highly consistent result, with a coefficient of −0.237 (significant at the 1% level) and a maximum standardized bias of 5.9%. In all three matching specifications, the maximum standardized bias for covariates remains well below the critical value of 10%, indicating that the matching procedures successfully balanced the observable characteristics between the treatment and control groups and effectively mitigated potential self-selection bias. Although the estimated coefficient magnitudes vary slightly due to the different weighting principles of each algorithm, the direction, significance, and qualitative conclusion are consistently aligned. These robust results collectively reinforce the core finding that the RSTI is associated with a reduction in pesticide expenditure. Detailed robustness test results are presented in Table 4.

**Table 4:** Robustness test results.

**Variable**	**Model 3**	**Model 4**	**Model 5**	**Model 6**
	**Pesticide reduction**	**Pesticide reduction**	**Pesticide reduction**	**Pesticide reduction**
RSTI	−0.298^***^	−0.506^***^	−0.236^***^	−0.237^***^
	(0.079)	(0.084)	(0.068)	(0.069)
Control variables	Yes	Yes	Yes	Yes
Sample size	3,524	2,364	4,265	4,266
Adjusted R^2^	0.0441	0.0464	0.029	0.029

Values in parentheses represent standard errors. ^***^p < 0.01.

However, it is important to note that while propensity score matching (PSM) approximates a randomized experiment by balancing observed covariates, its validity rests critically on the conditional independence assumption. This implies that PSM cannot address fundamental endogeneity concerns arising from unobserved confounders or reverse causality. Therefore, although our PSM analysis supports the robustness of the findings against observable selection bias, future research employing strategies such as instrumental variables would help further strengthen causal inference.

### 3.4 Heterogeneity test

To gain a deeper understanding of the impact of RSTI on pesticide reduction and to elucidate the differences among diverse subgroups, a heterogeneity test was conducted (Table 5). Specifically, the study employed alternative income stratification criteria, such as quartiles, to divide the sample. As well as plains and non-plains areas. Each of these subpopulations was individually assessed for significant disparities in the relationship between RSTI and pesticide reduction. The results of this heterogeneity analysis will provide crucial information for guiding the selection of subsequent analytical methodologies and ensuring the accuracy and reliability of the research outcomes.

**Table 5:** Heterogeneity test results: income level.

**Variable**	**Model 7**	**Model 8**	**Model 9**	**Model 10**
	**Pesticide reduction**	**Pesticide reduction**	**Pesticide reduction**	**Pesticide reduction**
RSTI	−0.216	−0.601^***^	0.099	−0.236
	(0.134)	(0.130)	(0.136)	(0.151)
Control variables	Yes	Yes	Yes	Yes
Sample size	1,067	1,063	1,065	1,059
Adjusted R^2^	0.0350	0.0505	0.0396	0.0076

Values in parentheses represent standard errors. ^***^p < 0.01.

#### 3.4.1 Heterogeneity analysis of different income levels

To examine the heterogeneous effects of the intervention across households with varying economic levels, the sample was divided into four income tiers: lowest income, lower-middle income, upper-middle income, and highest income, and analyzed accordingly. The quantile regression results reveal a clear pattern, indicating a significant non-linear relationship between the RSTI and pesticide cost per unit area. The most prominent finding is that the intervention produced a significant and negative effect only within the lower-middle-income group (Model 8, coefficient = −0.601, *p* < 0.01), meaning that the RSTI led to an approximately 45% reduction in pesticide cost per unit area for this group. This suggests that for this specific segment, the adoption of sanitary toilets is associated with a significant decrease in pesticide expenditure intensity. In contrast, the estimated coefficients for the lowest-income, upper-middle-income, and highest-income groups (Models 7, 9, and 10) are statistically insignificant, indicating that the intervention had limited effect in these groups.

#### 3.4.2 Heterogeneity analysis of terrain types

Terrain stands as a pivotal factor influencing agricultural production, farmers' lifestyles, and rural environments. In our total sample (*N* = 4,277), approximately 48.4% of households were located in plain areas, while 51.6% resided in non-plain areas, indicating a relatively balanced distribution across the two terrain types. To delve into whether these geographic disparities influence the relationship between RSTI and pesticide use, we conducted separate regression analyses on the plains and non-plains subsamples (Models 11–12). The findings revealed that, among residents in plain areas, the RSTI did not exert a significant impact on pesticide reduction (Model 11). In stark contrast, among residents in non-plain areas, the interventions exhibited a notable and significant effect on pesticide reduction (Model 12). Heterogeneity results by terrain type are reported in Table 6.

**Table 6:** Heterogeneity test results: terrain type.

**Variable**	**Model 11**	**Model 12**
	**Pesticide reduction**	**Pesticide reduction**
RSTI	0.137	−0.634^***^
	(0.110)	(0.085)
Control variables	Yes	Yes
Sample size	2,069	2,208
Adjusted R^2^	0.025	0.042

Values in parentheses represent standard errors. ^***^p < 0.01.

### 3.5 Regression analysis of the mediating effect of health literacy

Table 7 presents the results of the analysis examining the mediating role of health literacy. In the baseline mediation model, Model 13 treats health literacy as the dependent variable to assess the relationship between RSTI and health literacy. The results indicate that RSTI has a significantly positive effect on health literacy (coefficient = 0.096, *p* < 0.01), suggesting that the intervention effectively enhances residents' health knowledge, consistent with theoretical expectations. Model 14 incorporates health literacy into the regression analysis of pesticide use behavior. The findings show that health literacy significantly contributes to reducing pesticide use (coefficient = −0.128, *p* < 0.05). Moreover, after controlling for health literacy, the direct effect coefficient of RSTI decreases from −0.237 in the baseline model to −0.225. This suggests that health literacy partially mediates the relationship—i.e., the intervention not only directly promotes pesticide use reduction but also indirectly facilitates this behavioral change by improving residents' health literacy.

**Table 7:** Mediation effect analysis: health literacy as a mediator.

**Variable**	**Model 13**	**Model 14**	**Model 15**	**Model 16**
	**Health literacy**	**Pesticide reduction**	**Health literacy**	**Pesticide reduction**
RSTI	0.096^***^	−0.225^***^	0.098^***^	−0.225^***^
	(0.017)	(0.069)	(0.017)	(0.069)
Health literacy		−0.128^**^		−0.128^**^
		(0.062)		(0.062)
Control variables	Yes	Yes	Yes	Yes
Sample size	4,277	4,277	4,277	4,277
Adjusted R^2^	0.037	0.029	0.036	0.030

Values in parentheses represent standard errors. ^***^p < 0.01, ^**^p < 0.05.

Building on the baseline analysis, we further considered the potential conceptual and functional overlap between health literacy and educational attainment. Educational attainment reflects farmers' long-term accumulated cognitive foundation and learning ability, whereas health literacy in this study captures short-term behavioral changes, specifically farmers' initiative in acquiring health knowledge and enhancing health awareness under the rural sanitation toilet intervention and accompanying health education. In testing the mediation mechanism, the overlap between these two pathways could obscure the identification of the independent mediating effect of health literacy. To address this concern, we re-estimated the mediation model after excluding the educational attainment variable as a robustness check. The results are reported in Models 15 and 16.

Model 15 re-examines the relationship between RSTI and health literacy. The results show that RSTI continues to exert a significantly positive effect on health literacy (coefficient = 0.098, *p* < 0.01), closely mirroring the findings of Model 13. This indicates that the impact of RSTI on health literacy is robust, regardless of whether educational attainment is controlled. Model 16 further tests the mediation framework after excluding educational attainment. The results show that health literacy still significantly promotes the reduction of pesticide use (coefficient = −0.128, *p* < 0.05). Additionally, the direct effect of RSTI on pesticide reduction remains significant and stable at −0.225 (*p* < 0.01), comparable to the estimate in Model 14.

Overall, both the baseline model and the supplementary model excluding educational attainment consistently confirm that health literacy partially mediates the effect of RSTI on pesticide use reduction. This robustness check further supports the role of health literacy as an independent mediating variable, providing more rigorous empirical evidence for Hypothesis H2.

To ensure the rationality and rigor of the tests, the Bootstrap method was employed to re-examine the mediating effect of health literacy. When the 95% confidence interval excludes zero, it is considered that the indirect or direct effects are significant. The data presented in Table 8 reveal that both the mediating and direct effects along the mediating pathway were significant, implying that health literacy served as a partial mediator between the RSTI and pesticide reduction. More specifically, the RSTI indirectly facilitated pesticide reduction by enhancing the health literacy of farmers. This finding provides additional empirical support for H2.

**Table 8:** Bootstrap test for mediation effect.

	**Observed coef**.	**Bootstrap std. err**.	***Z***	***P* > |*Z*|**	Normal based [95% conf. interval]	
_bs_1	−0.0122116	0.0061282	−1.99	0.046	−0.0242227	−0.0002005
_bs_2	−0.2249708	0.0640398	−3.51	0.000	−0.3504866	−0.0994550

Referring to the test proposed by Wen and Ye (32).

## 4 Discussion

**Figure 2:**
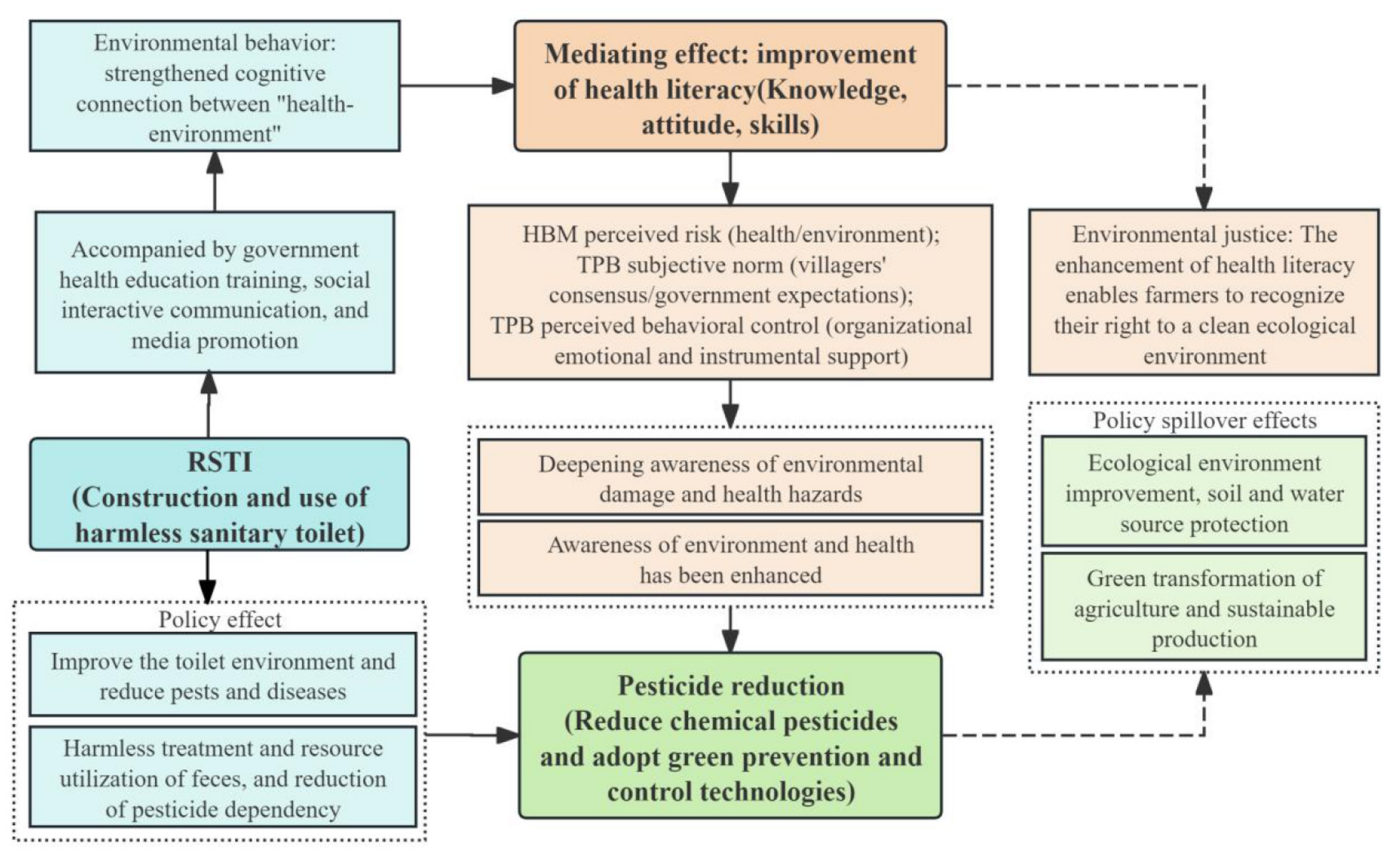
The Pathway from RSTI to pesticide reduction: the mediating role of health literacy.

This study, based on data from 4,277 rural households across ten provinces in China, explores the impact of RSTI—represented by non-hazardous sanitary toilets—on pesticide use behavior, with a specific focus on the mediating role of health literacy. The core findings are summarized in Figure 2, a schematic diagram that illustrates the mechanisms through which RSTI influences pesticide reduction via both direct and indirect pathways (mediated by health literacy), situated within a broader context of supporting factors and policy spillovers. The following sections provide a detailed discussion of these findings.

The analytical results indicate that RSTI significantly reduces the quantity of pesticides used. This conclusion remains robust even after excluding autonomous regions and economically developed provinces. Multiple robustness checks conducted through propensity score matching (including nearest neighbor matching, kernel matching, and radius matching) further support the statistical significance of this association. These results confirm that RSTI can effectively promote the reduction of agricultural pesticide use. This finding not only validates the theory of spillover effects, whereby improved sanitation facilities can enhance beneficiaries' confidence in and commitment to sustainable development (33), but also offers a fresh perspective on the connection between public health policies and agricultural sustainability.

Income heterogeneity analysis shows that the pesticide reduction effect of RSTI measures is only significantly promoted for the lower-middle-income group, with no significant effects observed in other income groups. Integrating theories of environmental justice and the Theory of Planned Behavior (TPB) helps explain this pattern. This particular group often faces dual deficiencies in access to environmental services and health information, and is typically more sensitive to economic costs and health outcomes, making them more susceptible to health shocks (34) and demonstrating the “optimal transformative capacity” in responding to public policy interventions (35). For this group, RSTI not only improves basic sanitary conditions but also, by enhancing health literacy, strengthens their perception of pesticide hazards (“perceived severity” in the Health Belief Model), thereby motivating more active adoption of pesticide-reduction practices. The non-significant effect for the lowest-income group may be influenced by a “livelihood-first” logic, where concerns over yield and income stability make it difficult to prioritize non-productive interventions. For upper-middle-income and highest-income groups, pesticide decisions are often dictated by standardized protocols within large-scale, market-oriented production systems, where the influence of individual health consciousness is relatively limited.

Geographical heterogeneity analysis finds that the pesticide reduction effect of RSTI is significant in non-plain areas but not in plain areas. This discrepancy can be explained from three perspectives: cultural-cognitive, agricultural structure, and policy implementation. Cognitively, farmers in plain areas are exposed to more diverse agricultural extension and market information, making the health education accompanying RSTI prone to being diluted. In non-plain areas, information dissemination relies more heavily on community networks and personal demonstration; thus, RSTI, as a visible public intervention, is more effective in stimulating perceived benefits related to the “health-environment” link, subsequently fostering behavioral change through community norms. Regarding agricultural structure, plain areas are characterized by large-scale, mechanized agriculture where pesticide decisions are subordinate to the logic of scaled production and are less likely to be influenced by sanitation improvements (36). In contrast, non-plain areas feature small-scale subsistence farming with closer links between daily life and production, making hygiene behaviors more likely to extend to field management. In terms of policy implementation, the high cost of upgrading sanitary facilities may lead to these measures being overlooked in rural non-plain areas, potentially exacerbating existing inequalities (37). Furthermore, the baseline coverage of sanitary facilities is generally higher in plain areas [e.g., in rural Zhejiang, coverage ranges from 10%−35% in plains vs. 1.7%−5% in mountainous areas (38)]. This higher baseline may diminish the marginal health perception gains from new interventions, thereby curbing behavioral spillover effects.

Mediation analysis indicates that health literacy plays a partial mediating role in the relationship between RSTI and pesticide reduction. This finding suggests that through mechanisms such as accompanying publicity and community interaction, RSTI can enhance farmers' health and risk awareness, thereby helping them better understand the long-term health and ecological hazards of pesticides and shaping their willingness to reduce pesticide use. While improved health literacy represents an important pathway through which the intervention influences behavior, it is not the sole mechanism. This distinction from a full mediation effect highlights the complexity of behavioral spillover from public health to agricultural practices. This mechanism reveals that public health interventions can not only directly improve environmental hygiene but also, by empowering individual cognition, stimulate pro-environmental behaviors in agricultural production.

The findings of this study resonate with and extend recent international literature focusing on sanitation, health literacy, and agricultural practices. Evidence from South Asia and Africa suggests the cross-contextual universality of the cognitive mediation pathway from sanitation improvement to pro-environmental agricultural behavior. Research from Malaysia indicates that information on pesticide hazards and awareness of environmental impacts influence pesticide use behavior (39); studies from India support the role of policy-backed initiatives in behavioral change, showing that government promotion of biopesticide use can enhance farmers' perceived behavioral control (40); evidence from Ethiopia links access to basic sanitation services with health risk perception and protective behaviors (41); research in Kenya confirms that social and behavior change communication can reduce pesticide risks (42); a study from Thailand suggests health literacy is influenced by socio-demographic and personal protection factors, indicating interventions must be adapted to local contexts (43); a cluster-randomized trial in Uganda demonstrates that educational interventions can significantly improve knowledge, attitudes, and practices related to pesticides, reducing exposure risks (44); research from Ghana provides further evidence at the eco-health level, showing that farmers' awareness of pesticide water pollution directly affects their risk perception of consuming contaminated fish, ultimately linking to their pesticide use behavior (45).

In summary, this study, through large-scale empirical evidence from China, links the public health intervention of “RSTI” with the agricultural environmental policy goal of “pesticide reduction,” validating the cognitive spillover pathway from sanitation infrastructure to pesticide reduction. By explicitly establishing and testing a mediation model via health literacy on a nationally representative sample, this research enriches the literature on the relationship between rural sanitation infrastructure and agricultural sustainability. It offers a new perspective for understanding how public health policies can influence agricultural sustainability and provides a replicable analytical framework for similar assessments in other developing agrarian economies. By reframing RSTI from a public health issue to a tool for environmental governance, this study broadens the research boundaries of policy spillover effects within the public administration discipline, contributing to a deeper understanding of the complex mechanisms underlying farmers' production decisions.

## 5 Conclusions

Leveraging data from the 2020 CRRS, this study conducts an empirical analysis of how the RSTI influences pesticide reduction and the mechanisms driving this relationship. The results indicate that RSTI significantly promotes pesticide reduction, a conclusion that remains robust after a series of sensitivity checks. Further analysis reveals that health literacy plays a partial mediating role in this process. Additionally, heterogeneity analysis shows that the pesticide-reduction effect of RSTI is concentrated mainly among the lower-middle-income group and is significantly associated with non-plain areas. This suggests that the environmental co-benefits of this policy are notably concentrated in specific groups and regions, providing important insights for designing integrated policies that coordinate public health and agricultural sustainable development.

Based on the findings, the following policy recommendations are proposed for decision-makers: first, given limited resources, a targeted investment strategy should be adopted. Priority should be given to promoting low-cost, locally adapted sanitary toilet technologies in non-plain areas where effects are significant. In plain areas, a “Sanitary Toilets and Precision Agriculture” program could be implemented, integrating health knowledge into existing agricultural extension systems. Second, establish a dedicated fund to support toilet upgrades for lower-middle-income households, and create bundled incentives pairing toilet upgrade with health and pesticide safety training. Eligible households could receive subsidies for green agricultural inputs and priority access to credit. Third, promote the deep integration of health literacy improvement and agricultural practices. Utilize community networks and diversified media to disseminate knowledge on green pest control, and build a service network that synergistically advances public health and agricultural transformation.

However, any targeted policy design based on these findings must remain vigilant against potential unintended consequences. Over-focusing on empirically effective groups and regions may neglect other vulnerable populations, creating new policy blind spots. Bundled incentives may trigger strategic compliance behaviors, weakening long-term effectiveness. If construction is emphasized over management, facilities may fall into disuse, undermining public trust in government projects. Therefore, it is recommended to establish a dynamic monitoring and evaluation mechanism. While implementing differentiated policies, universal access to basic sanitation services should be safeguarded, and the sustainable operation of infrastructure should be ensured.

Although this study provides new insights into the relationship between RSTI and pesticide reduction, several limitations must be acknowledged. First, the analysis is based on cross-sectional data, which limits the ability to infer strict causality or capture the long-term dynamic effects of such interventions. Although methods such as PSM were used to control for confounders, the possibility of omitted variable bias cannot be fully ruled out. Second, the measurement of key variables has limitations that may introduce measurement error. (1) The measure of pesticide reduction relies on farmers' self-reported pesticide expenditure per mu. While this indicator reflects changes in economic input, it cannot precisely capture differences in actual application quantity, toxicity levels, or frequency, and may be affected by recall error, social desirability bias, and potential pesticide price fluctuations. As a composite of “price × quantity,” changes in pesticide expenditure may stem from market price shifts (e.g., declines due to policy subsidies or industrial overcapacity, or increases due to supply shortages) rather than actual changes in application quantity, which may lead to measurement bias. (2) RSTI is measured as a binary variable that captures whether a household has a sanitary toilet meeting the unified policy standards (e.g., harmless treatment of feces, no leakage). While this indicator effectively reflects participation in the RSTI policy, it fails to capture nuanced variations beyond the basic quality threshold, such as toilet renovation mode, age, maintenance status, or frequency of use. These unmeasured dimensions may influence the strength of the policy's health-promoting and spillover effects, which could be addressed in future research with more detailed survey data. (3) The measurement of health literacy is based on a single question probing whether the individual actively seeks health knowledge. While this single item accurately captures the core behavioral dimension of proactive health information-seeking focused on in this study, it cannot fully capture the complex, multidimensional nature of health literacy, including its functional, interactive, and critical dimensions.

These limitations point to valuable directions for future research. First, a multi-source data validation framework could be constructed—for example, by integrating farmer self-reports with objective indicators such as pesticide sales records or soil/crop residue testing—to assess pesticide use behavior more comprehensively and accurately. Second, multidimensional, standardized health literacy scales (e.g., a short version of the HLS-EU-Q) should be directly incorporated into survey designs, or composite indicators developed, to measure this construct more precisely. Third, to address endogeneity concerns, future studies could adopt quasi-experimental designs or instrumental variable strategies using panel data. This would allow for a more reliable identification of the policy's causal, cumulative, and sustainability effects, while better controlling for individual heterogeneity and time trends. Building on such longitudinal data, future research could also explore how dynamic changes in the macro-environment (e.g., provincial economic conditions) and household income levels moderate the effects of the RSTI on pesticide reduction.

## Data Availability

The data analyzed in this study is subject to the following licenses/restrictions: The dataset (2020 China Rural Revitalization Survey, CRRS) is not publicly available due to the data management policy of the Rural Development Institute, Chinese Academy of Social Sciences. Access is only granted to qualified researchers upon reasonable request to the corresponding author, following the official application procedure specified at the CRRS data request portal, which restricts immediate or unrestricted access. Constraints on Usage Scope: The dataset is provided exclusively for academic research and statistical analysis purposes. It prohibits any use for commercial activities, or attempts to link the data with other information to identify individual respondents—consistent with ethical guidelines for protecting the privacy of survey participants. Requests to access these datasets should be directed to https://183.242.252.238:8081/database?rootId=CRRS&databaseId=8b48546db15e32624885960637f8420f.
